# Progress Toward Polio Eradication — Worldwide, January 2017–March 2019

**DOI:** 10.15585/mmwr.mm6820a3

**Published:** 2019-05-24

**Authors:** Sharon A. Greene, Jamal Ahmed, S. Deblina Datta, Cara C. Burns, Arshad Quddus, John F. Vertefeuille, Steven G.F. Wassilak

**Affiliations:** ^1^Epidemic Intelligence Service, CDC; ^2^Global Immunization Division, Center for Global Health, CDC; ^3^Polio Eradication Department, World Health Organization, Geneva, Switzerland; ^4^Division of Viral Diseases, National Center for Immunization and Respiratory Diseases, CDC.

Since the Global Polio Eradication Initiative (GPEI) began in 1988, transmission of wild poliovirus (WPV) has been interrupted in all countries except Afghanistan, Nigeria, and Pakistan. WPV type 2 (WPV2) was declared eradicated in 2015; WPV type 3 has not been detected since 2012 (*1*). After the certification of the eradication of WPV2, a global switch from trivalent oral poliovirus vaccine (tOPV, containing vaccine virus types 1, 2, and 3) to bivalent oral poliovirus vaccine (bOPV, containing types 1 and 3) was completed in April 2016. Nigeria last reported WPV type 1 (WPV1) cases in 2016. This report describes global progress toward poliomyelitis eradication during January 1, 2017–March 31, 2019, and updates previous reports (*1*,*2*). Afghanistan and Pakistan reported their lowest annual number of WPV cases (22) in 2017; however, 33 WPV1 cases were reported in 2018. During January–March 2019 (as of May 3), 12 WPV1 cases had been reported worldwide, four more than the eight reported during the corresponding period in 2018. The occurrence of polio cases caused by circulating vaccine-derived poliovirus (cVDPV) is rare and occurs where oral poliovirus vaccine (OPV) coverage has been low and vaccine virus reverts to neurovirulence (*3*). Eight countries (Democratic Republic of the Congo [DRC], Indonesia, Mozambique, Niger, Nigeria, Papua New Guinea, Somalia, and Syria) reported 210 cVDPV cases during 2017–2019 (as of May 3). Reaching children during supplemental immunization activities (SIAs), accessing mobile populations at high risk, and variations in surveillance performance represent ongoing challenges. Innovative efforts to vaccinate every child and strengthen coordination efforts between Afghanistan and Pakistan will help achieve eradication. For cVDPV outbreak responses to promptly stop transmission, intensified programmatic improvements are needed to make the responses more effective and limit the risk for generating future outbreaks.

## Poliovirus Vaccination

Estimated global coverage with 3 doses of poliovirus vaccines (Pol3, mostly OPV) through routine immunization services among infants aged >1 year was 88% in 2017 (the most recent year for which data are available).* However, national coverage estimates often mask low coverage and poor SIA quality in a substantial number of subnational areas. In the countries with endemic WPV transmission, estimated national Pol3 coverage in 2017 was 60% in Afghanistan, 40% in Nigeria, and 75% in Pakistan (*4*–*6*).

In 2017, a total of 172 SIAs were conducted in five World Health Organization (WHO) regions, during which nearly 1.8 billion total OPV and inactivated poliovirus vaccine (IPV) doses were allocated for use; 161 SIAs were conducted in 2018, with approximately 1.7 billion bOPV and IPV doses. Inaccessible areas and the inability to reach all children in fully accessible areas continue to pose barriers to achieving higher coverage.

Since the global withdrawal of all type 2–containing OPV vaccines, countries experiencing confirmed cVDPV type 2 (cVDPV2) outbreaks have requested authorization from the WHO Director-General to release monovalent OPV type 2 (mOPV2) vaccine for use. In 2017, 59 million mOPV2 doses (3.2% of total OPV) were used for outbreak response; 107 million mOPV2 doses (6.5%) were used in 2018.

## Poliovirus Surveillance

The primary means for detecting WPV and cVDPV transmission is through surveillance for acute flaccid paralysis (AFP) among children aged <15 years, with laboratory testing of stool specimens by WHO-accredited laboratories within the Global Polio Laboratory Network (*7*,*8*). The performance of AFP surveillance is assessed through two principal indicators. The first indicator is achieving an annual nonpolio AFP detection rate of ≥1 case per 100,000 population aged <15 years in countries in the WHO regions certified as polio-free, or ≥2 in all other countries; this rate is considered sufficiently sensitive to detect a case of polio. The second indicator is the collection of adequate stool specimens (i.e., two stool specimens collected >24 hours apart, within 14 days of paralysis onset, with arrival at the laboratory in good condition [cool and without leakage or desiccation]) from ≥80% of reported AFP patients.

Among countries reporting WPV or cVDPV cases in 2017, Afghanistan and Pakistan met both surveillance performance indicators nationally; DRC and Syria did not. Among the nine countries reporting WPV or cVDPV cases in 2018, Afghanistan, Indonesia, Mozambique, Niger, Nigeria, Pakistan, and Somalia met both surveillance performance indicators nationally; DRC and Papua New Guinea did not. Even when performance indicators are met nationally, surveillance gaps at the subnational level pose an impediment to reliable surveillance data that are necessary to ascertain the absence of poliovirus transmission. In many countries at high risk, AFP surveillance is supplemented by environmental surveillance (the testing of sewage samples).

## Poliovirus Cases and Isolations

**Countries reporting WPV cases and isolations.** In 2017, 22 WPV1 cases were reported, including 14 (64%) in Afghanistan and eight (36%) in Pakistan. In 2018, 33 WPV1 cases were detected, including 21 (64%) in Afghanistan and 12 (36%) in Pakistan. No WPV cases have been identified in countries other than Afghanistan, Nigeria, and Pakistan since 2015; Nigeria last reported WPV1 cases in September 2016. During January 1–March 31, 2019, 12 WPV1 cases were confirmed; six were detected in Afghanistan and six in Pakistan (Figure).

**FIGURE:**
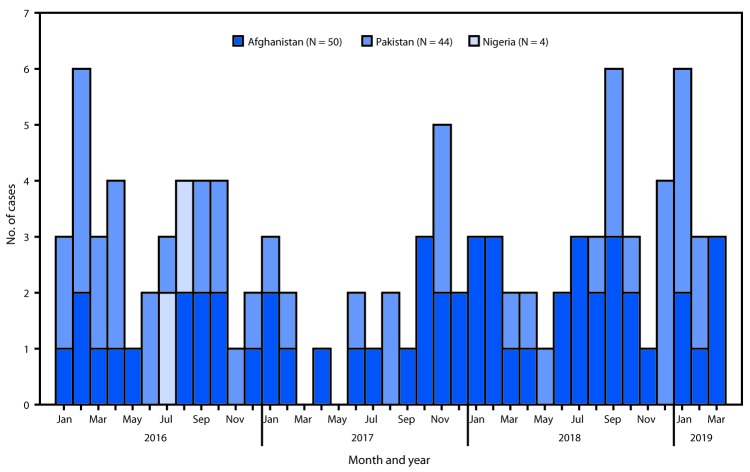
Number of cases of wild poliovirus, by country and month of onset — worldwide, January 2016–March 2019* * As of May 3, 2019.

Afghanistan reported 21 WPV1 cases in 14 districts in 2018, representing a 50% increase over the 14 cases reported in 2017 and a 55% increase in the number of affected districts. During January–March, 2019, six WPV1 cases were detected: one in each of two districts of Kandahar Province, two districts of Helmand Province, and two districts of Uruzgan Province, compared with a total of seven WPV1 cases reported in five districts of three provinces (Kandahar, Kunar, and Nangarhar) during the same period in 2018.

Pakistan confirmed 12 WPV1 cases in six districts in 2018, a 50% increase over the eight cases reported in 2017 and a 14% decrease from the seven districts that confirmed cases in 2017. During January–March, 2019, six WPV1 cases were detected: one in each of six districts located in three provinces (Khyber Pakhtunkhwa, Punjab, and Sindh), compared with only one case in Balochistan Province during the corresponding period in 2018.

Environmental surveillance is accounting for an increasing proportion of poliovirus detections worldwide. In Afghanistan, WPV1 was detected in 42 (13%) of 316 sewage samples collected at regular intervals in 2017 and 83 (24%) of 339 samples in 2018. In Pakistan, WPV1 was detected in 107 (17%) of 630 samples in 2017 and 141 (21%) of 677 samples in 2018 (Table 1). Genomic sequencing of poliovirus isolates from both environmental samples and confirmed AFP cases indicates multiple chains of transmission; five genetic clusters (groups of isolates sharing ≥95% of genetic relatedness) persisted during the reporting period in the core reservoirs along shared transnational population movement corridors between Afghanistan and Pakistan (*4*,*5*).

**TABLE 1:** Number of samples containing wild poliovirus type 1 (WPV1) detected through environmental surveillance — Afghanistan, Nigeria, and Pakistan, January 1, 2017–March 31, 2019*

Country	Surveillance period							
	2017		2018		Jan–Mar 2018		Jan–Mar 2019	
	No. of samples	WPV1 (%)	No. of samples	WPV1 (%)	No. of samples	WPV1 (%)	No. of samples	WPV1 (%)
Afghanistan	316	42 (13)	339	83 (24)	84	16 (19)	68	21 (31)
Nigeria	1,623	0 (0)	1,661	0 (0)	320	0 (0)	481	0 (0)
Pakistan	645	107 (17)	677	141 (21)	162	22 (14)	177	82 (46)

* Data as of May 3, 2019.

**Countries reporting cVDPV cases and isolations**. During January 2017–March 2019, cVDPV transmission was confirmed in nine countries. Two countries (Indonesia and Papua New Guinea) reported separate cVDPV type 1 (cVDPV1) circulation, with 27 AFP cases and seven positive environmental samples. Seven countries (DRC, Kenya, Mozambique, Niger, Nigeria, Somalia, and Syria) detected nine emergences of cVDPV2 with isolates from 176 AFP cases and 97 environmental samples. Nigeria reported no cVDPV isolates in 2017; however, in 2018, two cVDPV2 outbreaks were confirmed. One, centered in Sokoto, was detected through environmental surveillance; the other was initially detected in Jigawa State with subsequent detections in six other states and bordering Niger. An additional outbreak detected through environmental surveillance was confirmed in Bauchi State in March 2019. During 2018–2019 to date, 41 cVDPV2 cases have been detected in Nigeria and 10 in Niger. Since 2017, five independent cVDPV2 outbreaks, with 43 cases, have been reported in DRC. cVDPV2 transmission was detected from five AFP patients and three environmental surveillance sites in Somalia, and a genetically linked isolate was detected from an environmental surveillance site in Nairobi, Kenya. cVDPV type 3 transmission involving six AFP patients^†^ and 11 environmental samples was detected in Somalia (Table 2) (*9*).

**TABLE 2:** Number of poliovirus cases, by country — worldwide, January 1, 2017–March 31, 2019*

Countries	Period of onset							
	2017		2018		Jan–Mar 2018		Jan–Mar 2019	
	WPV1	cVDPV	WPV1	cVDPV	WPV1	cVDPV	WPV1	cVDPV
**Countries with endemic WPV1**								
Afghanistan	14	0	21	0	6	0	6	0
Nigeria	0	0	0	34	0	0	0	7
Pakistan	8	0	12	0	2	0	6	0
**Countries with reported cVDPV cases**								
Democratic Republic of the Congo	0	22	0	20	0	4	0	1
Indonesia	0	0	0	1	0	0	0	0
Mozambique	0	0	0	1	0	0	0	0
Niger	0	0	0	10	0	0	0	0
Papua New Guinea	0	0	0	26	0	0	0	0
Somalia	0	0	0	12^†^	0	0	0	1
Syria	0	74	0	0	0	0	0	0

**Abbreviations:** cVDPV = circulating vaccine-derived poliovirus; WPV1 = wild poliovirus type 1.

* Data as of May 3, 2019.

^†^ One patient with acute flaccid paralysis was coinfected with cVDPV type 2 and type 3.

## Discussion

No WPV1 cases have been detected in the WHO Africa Region in approximately 30 months. Continuing improvements in vaccinating children and surveillance in northeast Nigeria and other Lake Chad Basin countries suggest that WPV transmission might have been interrupted in the Africa Region. Additional analyses to assess surveillance sensitivity are needed to allow the Regional Commission for the Certification of Poliomyelitis Eradication to certify interruption.

For the first time since 2014, the number of annual WPV case reports in Afghanistan and Pakistan rose in 2018, in spite of targeted efforts to increase immunization in security-compromised districts, reduce vaccine refusal, and reach highly mobile populations. Genomic sequence analysis of isolates from AFP patients and environmental samples demonstrates not only persistent local transmission in reservoirs in both countries, but also ongoing transmission along two common corridors because of transnational population movements (*4*,*5*). Efforts are underway to enhance continuous immunization at border points in both countries. A ban on house-to-house vaccination in Kandahar Province since mid-2018 has negatively affected SIA effectiveness, and both countries’ programs continue to miss vaccinating a substantial number of eligible children in areas accessible to vaccinators. A need exists to comprehensively address local weaknesses in SIA implementation to increase population immunity and interrupt transmission.

Genetic characterization of the index isolates in nearly all cVDPV outbreaks suggested transmission for many years, indicating imprecise AFP surveillance systems. Indonesia and Papua New Guinea had last reported polio cases more than a decade ago; in both countries, there has been low routine immunization coverage before the emergence and spread of independent cVDPV1 (*10*). The multiple cVDPV2 outbreaks in DRC and Nigeria reflect the risk for cVDPV2 transmission where the number of SIAs had been insufficient or the quality of SIAs had been inadequate to increase type 2 immunity before the 2016 global switch from tOPV to bOPV (*9*). The SIAs in response to many of the cVDPV2 outbreaks have not been sufficiently timely or of sufficiently high quality to promptly interrupt transmission or to prevent the seeding of additional cVDPV emergences.

The persistence of WPV transmission and the number of cVDPV outbreaks underscore the need for country programs to more adequately assess and address the challenges to vaccinating all children. GPEI program goals for interruption of poliovirus transmission have been refocused through the development of the Polio Endgame Strategy 2019–2023.^§^ Adopting locally relevant, innovative approaches will increase effective implementation of the core strategies. In Afghanistan, goals include overcoming inaccessibility by renegotiating access to communities and engaging with local and religious leaders until house-to-house vaccination is reinstated. In Pakistan, increasing SIA quality will be addressed by more effectively engaging with communities to reduce the number of OPV refusals and to increase demand for immunization services, while also focusing on underperforming local areas. Unfortunately, rumors about the safety of OPV severely decreased the effectiveness of a recent SIA in Pakistan. A reassessment of risk communication and community engagement is ongoing, and a revised approach will be implemented in the most affected districts starting with the SIAs in June.

Periodic annual increases in the number of polio cases in the past have always been followed by a recommitment to interventions that work and innovative activities to access underimmunized populations. This commitment has enabled GPEI to reduce the number of countries with endemic poliovirus transmission to three since 2012 and the number of WPV cases to fewer than 100 every year since 2015. The critical objective is to reduce the number of areas with active transmission in Afghanistan and Pakistan simultaneously or within a short period. Revised emergency action plans for each country provide the roadmaps to further intensify and improve program operations and will need to be fully implemented in every locality to ensure the successful eradication of polio.

**Summary**

**What is already known about this topic?**

Wild poliovirus (WPV) transmission has not been interrupted in Afghanistan, Nigeria, and Pakistan. Rare circulating vaccine-derived poliovirus (cVDPV) outbreaks can occur in areas with low oral poliovirus vaccination coverage.

**What is added by this report?**

No WPV cases have been detected in Nigeria since 2016. WPV transmission has continued in Afghanistan and Pakistan in all previously identified reservoirs. The number and extent of cVDPV outbreaks increased in 2018. Countries with endemic polio have revised emergency action plans to innovate and intensify strategies to reach and vaccinate every child in underimmunized populations.

**What are the implications for public health practice?**

Successful implementation of locally relevant strategies in all areas will be essential to interrupting WPV transmission.

## Footnotes

* WHO-UNICEF estimates of Pol3 coverage. http://apps.who.int/immunization_monitoring/globalsummary/timeseries/tswucoveragepol3.html.

† One AFP patient detected in Somalia was coinfected with cVDPV type 2 and type 3.

§ World Health Organization. The Polio Endgame Strategy 2019–2023: Eradication, Integration, Containment and Certification. http://polioeradication.org/who-we-are/polio-endgame-strategy-2019-2023.
